# Red clover extract exerts antidiabetic and hypolipidemic effects in db/db mice

**DOI:** 10.3892/etm.2012.658

**Published:** 2012-08-09

**Authors:** LONGXIN QIU, TONG CHEN, FOJIN ZHONG, YAMIN HONG, LIMEI CHEN, HONG YE

**Affiliations:** 1 School of Life Sciences and; 2 Fujian Key Laboratory of Preventive Veterinary Medicine and Biotechnology, Longyan University, Longyan 364000, P.R. China

**Keywords:** red clover extract, type 2 diabetes mellitus, lipid homeostasis, peroxisome proliferator-activated receptors, fatty acid synthase

## Abstract

To investigate the effects of red clover extract on the blood glucose and lipid levels of type 2 diabetic db/db mice, male db/db mice were treated with this extract for a period of 5 weeks. The red clover extract had a significant effect on lowering the blood glucose levels of db/db mice. The serum triglyceride, serum total cholesterol, liver triglyceride and liver cholesterol levels for diabetic mice receiving red clover extract were significantly lower compared to those of the untreated diabetic mice. The mRNA expression of two target genes transcriptionally regulated by peroxisome proliferator-activated receptor (PPAR)γ was determined by quantitative real-time RT-PCR, and red clover extract was observed to significantly upregulate hepatic glucokinase and CD36 expression. Four target genes transcriptionally regulated by PPARα were also assayed, and red clover extract was observed to significantly downregulate hepatic apolipoprotein C3 expression whereas it had no significant effect on apolipoprotein A5, acetyl CoA oxidase and carnitine palmitoyl transferase-1 expression. In addition, hepatic mRNA expression of fatty acid synthase was also observed to be downregulated by red clover extract treatment. Thus, we conclude that red clover extract significantly improves the glucose and lipid homeostasis in db/db diabetic mice and that these effects are achieved at least in part by activating hepatic PPARα/γ and by inhibiting hepatic fatty acid synthase.

## Introduction

Plant extracts containing isoflavones have been the focus of numerous studies during the last decade due to their protective effects against menopausal symptoms and a variety of disorders, including cardiovascular disease, cancer, hyper-lipidemia and osteoporosis (1,2). In addition, the potential of dietary isoflavones in the prevention of diabetes mellitus has attracted increased attention among the public and in the medical community in recent years (2,3). Soy isoflavones were reported to be beneficial for correcting hyperglycemia and improving lipid profiles in streptozotocin (STZ)-induced diabetic rats (4) and obese Zucker rats (5). The intervention studies of patients with type 2 diabetes have reported favorable effects of soy isoflavones on glycated hemoglobin or insulin resistance (6,7). However, other studies have reported that soy isoflavone intake does not exert beneficial effects on patients with type 2 diabetes (8,9). Therefore, the effect of isoflavones on diabetes remains inconclusive and more investigations need to be performed using isoflavones from plants other than soy.

While soy isoflavones are the most studied isoflavones used in studies on diabetes, few data are available for red clover (*Trifolium pratense*) isoflavones. Isoflavones from red clover differ from soy; the principal isoflavones in red clover are biochanin A, formononetin, genistein and daidzein while those in soy consist solely of genistein and daidzein. We previously reported that red clover extract ameliorated dyslipidemia in STZ-induced type 1 diabetic mice, but did not correct hyperglycemia (10). However, whether red clover extract exerts antidiabetic and hypolipidemic effects in type 2 diabetic animals remains unclear.

Among the mechanisms whereby isoflavones ameliorate hyperglycemia and dyslipidemia, one may be the activation of peroxisome-proliferator activated receptors (PPARs), nuclear receptors that participate in cellular lipid homeostasis and insulin action (11). PPARγ ligands, like glitazones, are clinically used to treat type 2 diabetes as insulin-sensitizing drugs and PPARα ligands, like fibrates, are used to manage elevated blood lipid levels and type 2 diabetes as hypolipidemic agents. Isoflavones from red clover were demonstrated to be a potent PPARα/γ dual agonist, and among those isoflavones, biochanin A is a more potent PPARα/γ agonist than its metabolite genistein, and formononetin is also more potent than daidzein (12). Thus, further study on the antidiabetic effects and molecular mechanisms of red clover extract in type 2 diabetes animal models needs to be conducted. In the present study, using db/db mice as a model of type 2 diabetes, we aimed to determine whether red clover extract exerts antidiabetic and hypolipidemic effects in type 2 diabetic animals. We also investigated whether the PPARα/γ agonist mechanisms of red clover isoflavones are involved in the hyperglycemia and dyslipidemia improvement.

## Materials and methods

### Materials

Red clover extract was purchased from a common Chinese pharmacy and was standardized to 10% isoflavones (consisting of 10.2% formononetin, 9.6% biochanin A, 0.32% genistein and 0.08% daidzein).

### Animal experiments

All experiments were conducted according to protocols and guidelines approved by Longyan University Institutional Animal Care and Use Committee. db/db (BKS.Cg-m/Lepr^db^/J) mice were obtained from the Jackson Laboratory (Bar Harbor, ME, USA). All animals were maintained on a standard laboratory diet under a 12/12-h light/dark schedule. Male db/db mice, 7–8 weeks of age, were randomly divided into 3 experimental groups (each containing 6 animals): db/db mice, db/db + 10 mg/kg/day red clover extract, db/db + 50 mg/kg/day red clover extract. Red clover extract was administered orally in 0.5% sodium carboxymethyl cellulose (CMC) suspension and continued for 35 days.

### Determination of blood glucose and serum lipids

The blood glucose level was measured periodically throughout the experimental period using a glucometer (OneTouch Ultra; LifeScan, Inc., Milpitas, CA, USA). At the end of the red clover extract treatment, the mice were sacrificed and blood was collected by orbital sinus puncture. Serum triglycerides (TG) and total cholesterol (TC) were measured using commercial kits (Jiancheng, Nanjing, China).

### Liver lipid analyses

Liver lipid was extracted by chloroform/methanol. Briefly, pulverized liver was homogenized in PBS, then extracted with chloroform/methanol (2:1), dried overnight and resuspended in a solution of 60% butanol 40% Triton X-114/methanol (2:1). Liver total TG and cholesterol levels were measured using colorimetric assays (Jiancheng).

### Quantitative analyses of the mRNA expression by real-time PCR

Total RNA was isolated from tissues using the TRIzol reagent (Invitrogen, Carlsbad, CA, USA) according to the manufacturer’s instructions. Complementary DNA (cDNA) was synthesized from hepatic mRNA using RevertAid™ First Strand cDNA Synthesis kits (Fermentas, Vilnius, Lithuania). Hepatic acetyl CoA oxidase (ACO), carnitine palmitoyl transferase-1 (CPT-1), apolipoprotein A5 (APOA5), APOC3, sterol regulatory element binding protein 1c (SREBP-1c) and fatty acid synthase (FAS) mRNA were analyzed with the specific primers listed in Table I. Real-time polymerase chain reactions were assayed using the FastStart Universal SYBR-Green Master (Rox; Roche Applied Science, Mannheim, Germany). Each Ct value was normalized to 18S rRNA.

### Statistical analysis

Quantitative data are expressed as mean ± SEM. The Student’s t-test was used for pairwise comparisons and one-way ANOVA with Newman-Keuls multiple comparison test for multigroup analyses. P<0.05 was considered to indicate a statistically significant result.

## Results

### Red clover extract attenuates hyperglycemia in db/db mice by activating PPARγ

**Figure 1:**
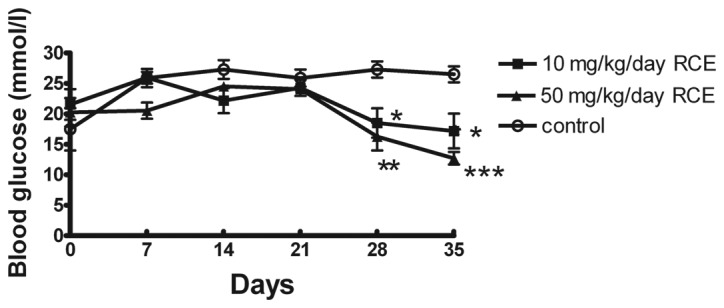
Effect of red clover extract treatment on blood glucose level of db/db diabetic mice. Mouse groups used were db/db, 10 mg/kg red clover extract (RCE)-treated db/db and 50 mg/kg RCE-treated db/db (n=6/group). The data are expressed as mean ± SEM, ^*^P<0.05; ^**^P<0.01; ^***^P<0.001.

To investigate the effect of red clover extract on the development of diabetes, we treated two groups of mice with red clover extract at the doses of 10 or 50 mg/kg/day, respectively, for 5 weeks. Fig. 1 shows the effect of red clover extract treatment on blood glucose level in db/db diabetic mice. After 4 weeks treatment with red clover extract, the blood glucose levels of the 10 and 50 mg/kg/day red clover extract-treated db/db mice were 18.5±2.4 and 16.3±2.3 mmol/l, respectively, compared with 27.3±1.3 mmol/l in the control untreated db/db mice (P<0.05 and P<0.01, respectively). In addition, after 5 weeks of treatment with red clover extract, the blood glucose levels of the 10 and 50 mg/kg/day red clover extract-treated db/db mice were 17.2±2.9 and 12.7±1.0 mmol/l, respectively, compared with 26.5±1.3 mmol/l in the control untreated db/db mice (P<0.05 and P<0.001, respectively). Our data suggest that red clover extract treatment attenuates hyperglycemia in type 2 diabetic animals.

**Figure 2:**
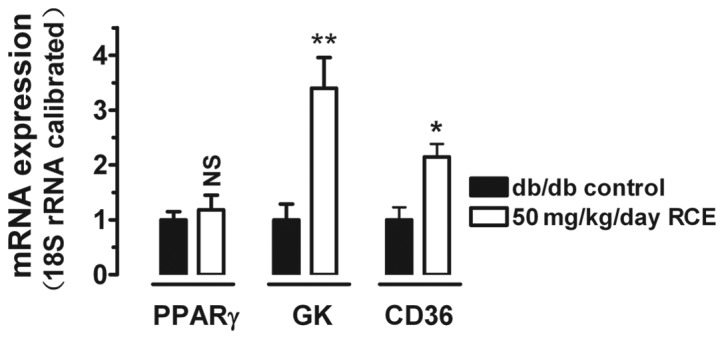
Hepatic mRNA expression of PPARγ, glucokinase and CD36 after RCE treatment in db/db mice. mRNA expression was analyzed by quantitative real-time RT-PCR. Values are expressed as mean ± SEM. ^*^P<0.05; ^**^P<0.01. n=4 for each group. GK, glucokinase; RCE, red clover extract.

Glucokinase, a key enzyme involved in the regulation of glucose metabolism, and CD36, a scavenger receptor involved in hepatic fatty acid uptake, are two hepatic genes regulated by PPARγ (13,14). To determine whether red clover extract attenuates hyperglycemia in diabetic mice by activating PPARγ, we analyzed the mRNA expression of PPARγ, glucokinase and CD36. After 5 weeks of red clover extract treatment, expression of glucokinase and CD36 was significantly upregulated (3.4-fold, P<0.01 and 2.1-fold, P<0.05) in 50 mg/kg/day red clover extract-treated diabetic mice (Fig. 2).

### Red clover extract regulates lipid homeostasis in db/db mice by activating hepatic PPARα and inhibiting hepatic FAS

**Figure 3:**
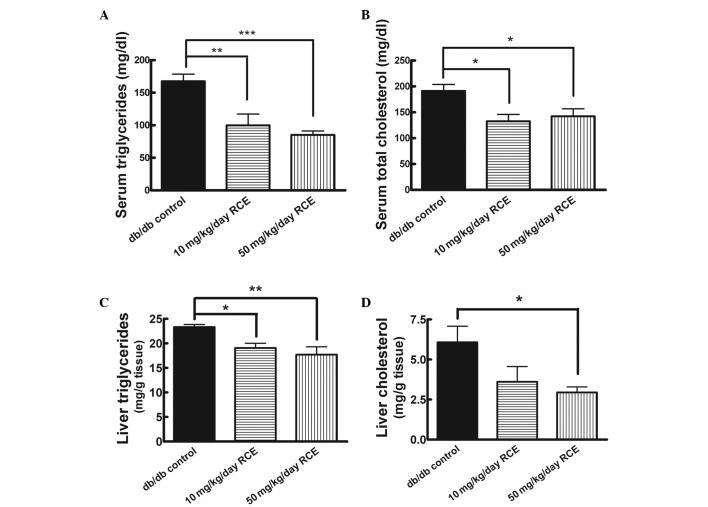
Effect of red clover extract treatment on serum and liver lipid profiles in db/db mice. Red clover extract (RCE) treatment significantly decreased the (A) serum triglyceride, (B) serum total cholesterol, (C) liver triglyceride and (D) liver cholesterol levels in db/db mice. n=6 for each group. Data are expressed as mean ± SEM. ^*^P<0.05; ^**^P<0.01; ^***^P<0.001.

To determine the effects of red clover extract on the overall lipid metabolism in the diabetic animals, we examined the levels of serum TG and TC in db/db mice with or without red clover extract treatment for a period of 5 weeks. Red clover extract-treated db/db mice had significantly lower blood TG (99.9±17.4 mg/dl for db/db + 10 mg/kg red clover extract vs. 167.5±11.0 mg/dl for db/db, P<0.01; 85.2±6.0 mg/dl for db/db + 50 mg/kg red clover extract vs. 167.5±11.0 mg/dl for db/db, P<0.001) in comparison with the untreated db/db mice (Fig. 3A). In addition, both 10 and 50 mg/kg/day of red clover extract treatment caused significant decreases in serum TC levels in db/db mice (132.7±13.3 mg/dl for db/db + 10 mg/kg red clover extract vs. 191.1±12.8 mg/dl for db/db, P<0.05; 142.2±14.7 mg/dl for db/db + 50 mg/kg red clover extract vs. 191.1±12.8 mg/dl for db/db, P<0.05; Fig. 3B). However, the TC lowering effect was not dose-dependent. Notably, no apparent difference in food consumption was observed in all the experimental groups (data not shown).

In addition, we investigated the effects of red clover extract on the liver lipid levels in db/db mice. As shown in Fig. 3C and D, 10 mg/kg/day and 50 mg/kg/day red clover extract treatment in db/db mice significantly reduced hepatic TG by ∼18.3 and 24.1%, respectively, (19.1±1.0 mg/g tissue for db/db + 10 mg/kg red clover extract vs. 23.3±0.5 mg/g tissue for db/db, P<0.05; 17.7±1.6 mg/g tissue for db/db + 50 mg/kg red clover extract vs. 23.3±0.5 mg/g tissue for db/db, P<0.01) and reduced hepatic cholesterol by ∼40.5 and 51.5%, respectively (3.6±0.9 mg/g tissue for db/db + 10 mg/kg red clover extract vs. 6.1±1.0 mg/g tissue for db/db, P>0.05; 2.9±0.3 mg/g tissue for db/db + 50 mg/kg red clover extract vs. 6.1±1.0 mg/g tissue for db/db, P<0.05).

**Figure 4:**
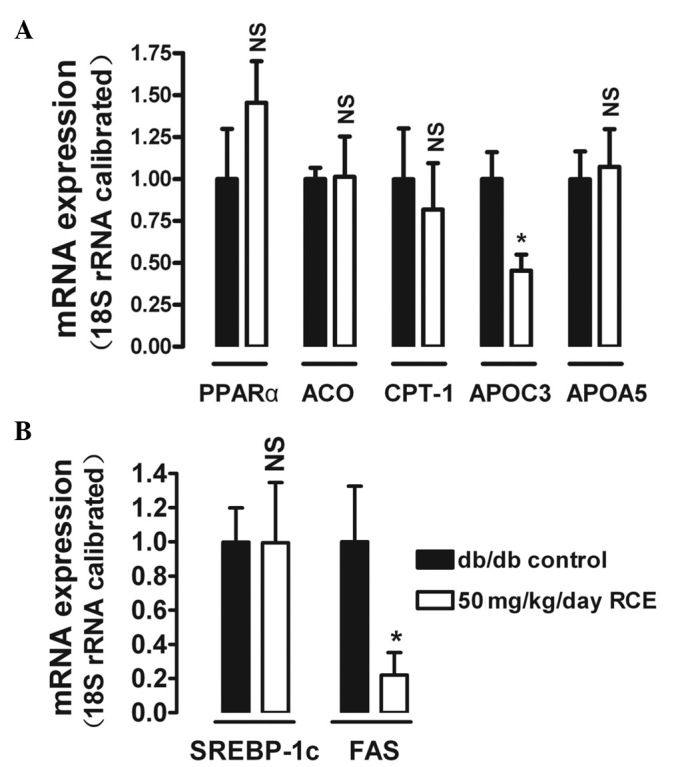
Hepatic mRNA expression of genes involved in lipid metabolism after RCE treatment in db/db mice. mRNA expression was analyzed by quantitative real-time RT-PCR. Values are expressed as mean ± SEM. ^*^P<0.05. n=4 for each group. RCE, red clover extract.

Isoflavones like biochanin A and formononetin are reported to be potent agonists of PPARα (12,15,16). To determine the effects of red clover extract treatment on certain target genes of PPARα in the liver, we analyzed hepatic ACO, CPT-1, APOC3 and APOA5 mRNA expression in db/db diabetic mice. After 5 weeks of red clover extract treatment, APOC3, a protein capable of inhibiting TG hydrolysis by lipoprotein lipase (LPL) was downregulated by 54.8% (P<0.05) in the 50 mg/kg red clover extract-treated diabetic mice (Fig. 4A). In contrast to APOC3, only slight and insignificant alterations were observed for APOA5, ACO and CPT-1.

The effect of red clover extract on fatty acid synthesis was also investigated. After 5 weeks of red clover extract treatment, the hepatic mRNA expression of FAS was significantly downregulated by 78% (P<0.05) in the 50 mg/kg red clover extract-treated diabetic mice (Fig. 4B). However, mRNA expression of SREBP-1c, a protein that regulates FAS expression, was not changed by red clover extract treatment.

## Discussion

*In vitro* studies have demonstrated that biochanin A, formononetin and genistein, three of the isoflavones from red clover are effective PPARγ agonists (12,15,16). In this study, we demonstrated that red clover extract treatment resulted in activation of known PPARγ-regulated genes in the liver *in vivo*. The activation of PPARγ improves insulin sensitivity, which maintains the blood glucose homeostasis (17). The glucokinase activation resulting from PPARγ activation also maintains the blood glucose homeostasis since glucokinase is a glucose sensor (14). Therefore, the PPARγ agonist property of red clover extract could partially explain its hypoglycemic mechanism. However, certain studies reported that purified isoflavones were ineffective at activating PPARγ whereas soy protein isolate was effective *in vivo* (18), which suggests that nonisoflavone phytochemicals or their metabolites are responsible for the activation of PPARγ. Therefore, whether biochanin A and formononetin or other nonisoflavone phyto-chemicals from red clover extract activate hepatic PPARγ in diabetic mice remains to be investigated.

The hypoglycemic effects of isoflavones remain controversial. Certain studies have reported that isoflavones are beneficial for correcting hyperglycemia in STZ-induced diabetic rats (4), obese Zucker rats (5) and in patients with type 2 diabetes (6,7). Certain other studies have reported that isoflavone intake does not exert anti-hyperglycemic effects on STZ-induced diabetic mice (10) and type 2 diabetes patients (8,9). In addition, there are certain debates upon the hypolipidemic effect of red clover isoflavones under various disease conditions. Several studies reported the effect of red clover extract on improving lipid profile (10,19–22) whereas other studies did not identify an effect (23,24). Our current data is consistent with certain previous studies (10) suggesting that red clover extract ameliorates dyslipidemia in diabetic animals. Furthermore, we demonstrated that red clover extract downregulated the hepatic mRNA expression of APOC3, a target gene transcriptionally regulated by PPARα. PPARα is an important metabolic nuclear receptor that regulates lipid metabolism through direct transcriptional control of genes involved in peroxisomal and mitochondrial β-oxidation pathways, fatty acid uptake, and TG catabolism (25). Hepatic activation of PPARα by its agonists, such as WY-14643, decreases blood TG levels by upregulating the expression of LPL and APOA5 and downregulating APOC3 (26,27). In the present study, however, the mRNA expression of APOA5, ACO and CPT-1, three other PPARα-regulated genes, was not altered by red clover extract treatment. The diversity of PPARα target gene expression following PPARα activation was also reported in certain other studies (10,28), yet the underlying molecular mechanism remains unclear.

FAS is the multifunctional protein that plays a central role in *de novo* fatty acid synthesis and in the long-term regulation of lipogenesis (29). The promoter region of FAS contains binding sites for the transcription factor called SREBP-1c (30). In the present study, we demonstrated that the hepatic mRNA expression of FAS in diabetic db/db mice was significantly downregulated by red clover extract treatment. However, SREBP-1c mRNA expression was not altered simultaneously. SREBP is synthesized as inactive precursors and the NH2 terminal domain of SREBP must be cleaved in a 2-step proteolytic process by site-1 (S1P) and site-2 (S2P) proteases to act as a transcription factor. This proteolytic release of SREBP stimulates lipid synthesis in hepatocytes and other cells (31). Notably, genistein treatment of HepG2 cells was found to decrease the expression of FAS but did not change the expression of SREBP-1 mRNA, likely via the downregulation of S1P expression and subsequent SREBP-1 proteolytic cleavage (32). Thus, whether red clover isoflavones reduce FAS expression by the same pathway remains to be elucidated.

To the best of our knowledge, the present study is the first to demonstrate the antidiabetic effect of red clover extract in type 2 diabetic animals. Red clover extract improves lipid homeostasis in type 2 diabetic animals. In the present study, we demonstrated the PPARα/γ agonist activities *in vivo* and elucidated the antidiabetic and hypolipidemic mechanism of red clover extract. We also demonstrated that red clover extract suppressed lipogenesis by inhibiting FAS activity. Our data indicate the benefit of a dietary supplement of red clover extract for diabetes patients.

## Figures and Tables

**Table I:** Primer sequences used for amplification of mRNA by real-time PCR.

Gene	Sequences
PPARα	Forward: 5′-AAGAGGGCTGAGCGTAGGT-3′
	Reverse: 5′-GGCCGGTTAAGACCAGACT-3′
APOC3	Forward: 5′-GTGTTGCAGATGTGCCTGTT-3′
	Reverse: 5′-GGAGGGGTGAAGACATGAGA-3′
APOA5	Forward: 5′-GAACGCTTGGTGACTGGAAT-3′
	Reverse: 5′-TCGCCTTACGTGTGAGTTTG-3′
ACO	Forward: 5′-CCACATATGACCCCAAGACC-3′
	Reverse: 5′-AGGCATGTAACCCGTAGCAC-3′
CPT-1	Forward: 5′-GTCAAGCCAGACGAAGAACA-3′
	Reverse: 5′-CGAGAAGACCTTGACCATAG-3′
FAS	Forward: 5′-TGCTCCCAGCTGCAGGC-3′
	Reverse: 5′-GCCCGGTAGCTCTGGGTGTA-3′
SREBP-1c	Forward: 5′-ATCGGCGCGGAAGCTGTCGGGGTAGCGTC-3′
	Reverse: 5′-ACTGTCTTGGTTGTTGATGAGCTGGAGCAT-3′
PPARγ	Forward: 5′-CAAACCCTTACCACGGTTGA-3′
	Reverse: 5′-CCATTGGGTCAGCTCTTGTGA-3′
Glucokinase	Forward: 5′-TGAGATGGATGTGGTGGCAA-3′
	Reverse: 5′-CATGCCGACCTCACATTGG-3′
CD36	Forward: 5′-TGTTCCTCGCCATGAAATGA-3′
	Reverse: 5′-GCTAGGCAGCATGGAACTTGA-3′
18S rRNA	Forward: 5′-CGACGACCCATTCGAACGTCT-3′
	Reverse: 5′-CTCTCCGGAATCGAACCCTGA-3′
